# Genome-Wide Characterization of R2R3-MYB Transcription Factors in Pitaya Reveals a R2R3-MYB Repressor *HuMYB1* Involved in Fruit Ripening through Regulation of Betalain Biosynthesis by Repressing Betalain Biosynthesis-Related Genes

**DOI:** 10.3390/cells10081949

**Published:** 2021-07-31

**Authors:** Fangfang Xie, Qingzhu Hua, Canbin Chen, Zhike Zhang, Rong Zhang, Jietang Zhao, Guibing Hu, Jianye Chen, Yonghua Qin

**Affiliations:** State Key Laboratory for Conservation and Utilization of Subtropical Agrobioresources/Guangdong Provincial Key Laboratory of Postharvest Science of Fruits and Vegetables/Key Laboratory of Biology and Genetic Improvement of Horticultural Crops, Ministry of Agriculture and Rural Affairs, College of Horticulture, South China Agricultural University, Guangzhou 510642, China; xiefangfang202012@163.com (F.X.); huaqingzhu@stu.scau.edu.cn (Q.H.); nnchencanbin@163.com (C.C.); poloky2@163.com (Z.Z.); r-zhang@scau.edu.cn (R.Z.); jtzhao@scau.edu.cn (J.Z.); guibing@scau.edu.cn (G.H.)

**Keywords:** pitaya, genome-wide, R2R3-MYB transcription factor, betalain biosynthesis, fruit ripening, repressor

## Abstract

The MYB (myeloblastosis) superfamily constitutes one of the most abundant transcription factors (TFs) regulating various biological processes in plants. However, the molecular characteristics and functions of MYB TFs in pitaya remain unclear. To date, no genome-wide characterization analysis of this gene family has been conducted in the Cactaceae species. In this study, 105 R2R3-MYB members were identified from the genome data of *Hylocereus undatus* and their conserved motifs, physiological and biochemical characteristics, chromosome locations, synteny relationship, gene structure and phylogeny were further analyzed. Expression analyses suggested that three up-regulated *HuMYBs* and twenty-two down-regulated *HuMYBs* were probably involved in fruit ripening of pitaya. Phylogenetic analyses of R2R3-MYB repressors showed that seven *HuMYBs* (*HuMYB1*, *HuMYB21*, *HuMYB48*, *HuMYB49*, *HuMYB72*, *HuMYB78* and *HuMYB101*) were in clades containing R2R3-MYB repressors. *HuMYB1* and *HuMYB21* were significantly down-regulated with the betalain accumulation during fruit ripening of ‘Guanhuahong’ pitaya (*H. monacanthus*). However, only *HuMYB1* had R2 and R3 repeats with C1, C2, C3 and C4 motifs. *HuMYB1* was localized exclusively to the nucleus and exhibited transcriptional inhibition capacities. Dual luciferase reporter assay demonstrated that *HuMYB1* inhibited the expression of betalain-related genes: *HuADH1*, *HuCYP76AD1-1* and *HuDODA1*. These results suggested that *HuMYB1* is a potential repressor of betalain biosynthesis during pitaya fruit ripening. Our results provide the first genome-wide analyses of the R2R3-MYB subfamily involved in pitaya betalain biosynthesis and will facilitate functional analysis of this gene family in the future.

## 1. Introduction

Transcription factors (TFs) play important roles in plant growth and development by controlling gene expression, suppressing translation or activating transcription of target genes, or interacting with other proteins. Generally, TFs are composed of at least four discrete domains: DNA-binding domain, nuclear localization signal, transcription-activation domain and oligomerization site [1]. The MYB family is one of the largest TF families known in eukaryotes and is defined by the presence of one to four highly conserved MYB repeats. The MYB repeat contains approximately 52 amino acids with three regularly spaced tryptophan, encoding three α-helices. The third helix of each repeat plays a recognition role in direct binding to DNA sequence and intercalating in the major groove [2]. Based on the MYB repeat number and the identity of the MYB repeats, MYB superfamily members are generally classified into four major subfamilies, namely, MYB-related (1R-MYB), R2R3-MYB (2R-MYB), R1R2R3-MYB (3R-MYB) and 4R-like MYB (4R-MYB) proteins. All four subfamilies are found in plants, representing the taxon with the highest diversity of MYB proteins. The smallest subfamily is the 4R-MYB group, whose members contain four R1/R2-like repeats in several plant genomes [3]. The 3R-MYB subfamily contains R1, R2 and R3 repeats and typically encoded by five genes in higher plant genomes. The 1R-MYB subfamily comprises proteins with a single or a partial MYB repeat and falls into several subclasses, including R3-type, R1/R2-type and the GARP family [4].

Most plant *MYB* genes encode proteins of the 2R-MYB class, which contain an R2 and an R3 domain at N terminal and an activation or repression motif at the C terminus. The R2R3-MYB subfamily probably evolved from an *R1-MYB* genes through duplication of an R1 repeat or from an *R1R2R3-MYB* gene through loss of the R1 repeat [4,5]. Subsequently, genome-wide duplication and tandem duplication events play important roles in the rapid expansion of R2R3-MYB members and its subfamily/clade-asymmetric and lineage-specific in land plants [6,7]. The first *MYB* gene, named *v-MYB*, was identified from avian myeloblastosis virus (AMV) [8]. Meanwhile, the first plant MYB gene, named *C1*, was isolated from *Zea mays* and encoded a c-MYB-like TF involved in anthocyanin pigmentation [9]. With the growing number of fully sequenced plant genomes, great progress has been made in identification of *R2R3-MYB* genes in recent years. Based on their well conserved MYB domains, R2R3-MYB families have been annotated genome-wide in many plant such as Arabidopsis (*Arabidopsis thaliana*, 126 members) [3], grape *(Vitis vinifera*, 117 members) [10], apple (*Malus × domestica*, 222 members) [11], kiwifruit (*Actinidia chinensis*, 93 members) [12], pineapple (*Ananas comosus*, 87 members) [13], watermelon (*Citrullus lanatus*, 162 members) [14], banana (*Musa acuminata*, 285 members) [15] and beet (*Beta vulgaris*, 70 members) [16]. These R2R3-MYB subfamilies are involved in the regulation of plant development, cell differentiation, primary and secondary metabolism and stress responses. R2R3-MYBs associated with flavonoid production or suppression can be identified by phylogenetic analyses. In Arabidopsis, the members of subgroup 4–7 could regulate the flavonoid biosynthesis [3]. *BvMYB1* was found to activate the expression levels of *CYP76AD1* and *DODA1* resulting in betalain accumulation in beet [17]. However, *BvMYB1* is clustered in subgroup 6 responsible for anthocyanin biosynthesis [7]. Moreover, there is no study about the R2R3-MYB subfamily in pitaya and no information is available about repressor involved in betalain biosynthesis.

Pitaya, belonging to *Hylocereus* in the Cactaceae family of Caryophyllales, is mainly classified into three groups including *H. undatus* (red peel with white pulp), *H. monacanthus* or *H. polyrhizus* (red peel with red pulp) and *H. megalanthus* or *Selenicereus megalanthus* (yellow peel with white pulp) [18]. Pitaya is the only edible fruit with abundant betalains that has been commercially produced at a large scale. Betalains are vacuole-localized, water-soluble and nitrogen-containing pigment derived from the L-tyrosine and divided into red-violet betacyanins and orange-yellow betaxanthins [19]. Four key enzymes are involved in betalain biosynthesis: (i) ADH (arogenate dehydrogenase), catalyzing the tyrosine (Tyr) biosynthesis [20]; (ii) CYP76AD1 (cytochrome P450), catalyzing the hydroxylation of tyrosine to DOPA and the subsequent oxidation of DOPA to cyclo-DOPA [21]; (iii) DODA (4,5-DOPA dioxygenase extradiol), catalyzing the L-DOPA to seco-DOPA and then quickly converting to betalamic acid spontaneously [22]; and (iv) GT (glucosyltransferase), catalyzing cyclo-DOPA to betanin [23]. As important natural colorants, betalains provide nutrients and health benefits for humans by increasing the antioxidant properties [24,25]. Betalain accumulation is an important index and parameter for measuring harvest time and fruit quality in pitaya. The color of pitaya peels and pulps is determined by betalain biosynthesis and accumulation. In this study, the genome-wide identification and characterization of *R2R3-MYB* genes were performed to screen candidate genes related to betalain biosynthesis using available genomic information. The aim of the present study is to identify and validate the *R2R3-MYB* involved in pitaya betalain biosynthesis and candidate *R2R3-MYB* genes can be used for genetic improvement of pitaya.

## 2. Materials and Methods

### 2.1. Identification and Sequence Analyses of Pitaya R2R3-MYB Subfamily

Plot hidden Markov model (HMM) profile of MYB DNA-binding domain (PF00249) was downloaded from Pfam (https://pfam.xfam.org/, accessed on 8 September 2019) [26] to identify *MYB* genes from pitaya genome [27]. To ensure the presence of the core MYB domains, the putative MYB sequences were further examined using Pfam and SMART (https://smart.embl-heidelberg.de/) (accessed on 27 March 2020). The sequences with two MYB domains were considered to be the final *R2R3-MYB* gene family members.

The DNA-binding domains of R2R3-MYB proteins were aligned by Cluster X software and shown by WEBLOGO online tool (https://weblogo.berkeley.edu/logo.cgi) (accessed on 27 March 2020). The conserved motifs of complete amino acid sequences were analyzed by Multiple Em for Motif Elicitation (https://meme-suite.org/) (accessed on 26 March 2020). The exon/intron organizations of 105 pitaya *R2R3-MYB* genes were drawn by TBtools software [28]. The physiological and biochemical characteristics of pitaya R2R3-MYB proteins were analyzed using the ExPASy (https://www.expasy.org/) (accessed on 27 March 2020).

### 2.2. Chromosomal Locations and Synteny Analyses of Pitaya R2R3-MYB Members

The MapChart software was used to draw the location sites of *HuMYBs* according to their physical positions. The duplication pattern for each *HuMYBs* was analyzed by Multiple Collonearity Scan toolkit (MCscanX) software following the operation manual [29]. The synteny relationship of orthologous *R2R3-MYB* genes between pitaya, beet and Arabidopsis was shown by TBtools software [28].

### 2.3. Phylogenetic Analyses of Pitaya R2R3-MYB Members

The evolutionary trees of pitaya, beet and Arabidopsis R2R3-MYB proteins were constructed using the Maximum likelihood method (ML) in MEGA 7 with 1000 bootstrap replicates. The phylogenetic trees of 105 pitaya R2R3-MYB proteins and R2R3-MYB repressors were performed by Neighbor-joining method (NJ) method. All phylogenetic trees were exhibited by EVOLVIEW online tool (https://www.evolgenius.info/evolview/) (accessed on 2 April 2020).

### 2.4. Plant Materials

‘Guanhuahong’ pitaya (red peel and green scales with red pulp, *H. monacanthus*) from the orchard of Jinsuinong (Zhongluotan Village, Guangzhou City, Guangdong Province, China) were used as materials. Fruits of ‘Guanhuahong’ pitaya were collected on the 14th, 17th, 19th, 23rd, 25th, 27th and 32nd day after flowering (DAF). All samples were frozen in liquid nitrogen immediately and stored at −80 ℃ until future analyses.

### 2.5. Measurement of Betalain Contents

Betalains were extracted and measured following our previously described method [30]. In brief, 0.5 g freeze-dried pitaya pulp powder were extracted with 5 mL 80% aqueous methanol (*v*/*v*) solution by sonication for 10 min and stirred for 20 min in dark at room temperature. After centrifuging at 5000× *g* for 15 min, the residues were subjected to a similar second extraction. The supernatants were measured through spectrophotometry (Infinite M200, Tecan Co., Ltd, Shanghai, China) at 478 nm for betaxanthins and 538 nm for betacyanins. All determinations were performed in three biological repetition with three technical replicates.

### 2.6. Gene Expression Analyses and Cloning

Transcriptome data of four fruit developmental stages (17th, 23rd, 25th and 32nd) (PRJNA704510) were used to draw the transcript abundance of 2R-MYB repressors by TBtools software. The primers were designed in NCBI (https://www.ncbi.nlm.nih.gov/tools/primer-blast/, accessed on 19 May 2020). Total RNA was isolated using the EASYspin Plus Complex Plant RNA Kit (RN53) (Aidlab Biotechnology, Beijing, China) according to the manufacturer’s protocol. Single-stranded cDNA was synthesized using the PrimeScript™ RT Reagent Kit with gDNA Eraser (TaKaRa, Shiga, Japan). qRT-PCR was performed with an CFX384-Real-Time System (C1000 Touch Thermal Cycler, Bio-Rad, Irvine, CA, USA) using the RealUniversal Color PreMix (SYBR Green) (TIANGEN, Beijing, China) with specific primers (Table S1). The *Actin (1)* was used as internal control for gene expression analyses [31]. All experiments were repeated in technical replicates. The relative expression levels of each transcript were calculated using the comparative Ct method [32].

The full-length coding sequences of *HuMYBs* were cloned using I-5^TM^ 2× High-Fidelity Master Mix (MCLAB, San Francisco, CA, USA) with specific primers (Table S1). Alignment of the deduced protein sequences was performed by Clustal X and GeneDoc software.

### 2.7. Subcellular Localization Analyses

Full-length coding sequences of *HuMYB1* were inserted into the pGreen-35S-GFP vector (primers are listed in Table S1). Subsequently, pGreen-35S-HuMYB1-GFP and pGreen-35S-GFP were separately transformed into *Agrobacterium tumefaciens* strain GV3101 (pSoup-p19) (Coolaber, Beijing, China) and infiltrated into *Nicotiana benthamiana* leaves. Two days after infiltration, leaf protoplasts were isolated according to Lai et al. [33] and the transient expression of GFP was observed using a fluorescence microscope (ZEISS LCM-800, Carl Zeiss, Oberkochen, Germany).

### 2.8. Transcriptional Activation Analyses in Yeast Cells

Full-length of *HuMYB1* were inserted into the pGBKT7 vector (primers are listed in Table S1). Then, the pGBKT7-HuMYB1, positive (pGBKT7-53 + pGADT7-T-antigen) and negative (pGBKT7) controls were separately transformed into yeast strain GoldY2H (Coolaber, Beijing, China) and grown on minimal medium plates without tryptophan (SD/-Trp) or without tryptophan, histidine and adenine (SD/-Trp-His-Ade). The transactivation activity of HuMYB1 protein was evaluated according to their growth status after 3 days in 30 ℃ and confirmed by incubating with X-α-galactosidase (X-α-Gal) for 30 min.

### 2.9. Dual-Luciferase Transient Expression Assay

DNA was isolated according to the protocol of Plant DNA Extraction Kit (DN14) (Aidlab, Beijing, China) and RNA was removed with Ribonuclease A (RNase A) (Takara Biomedical Technology Co., Ltd, Beijing, China). Promoter sequences were cloned using Seq Amp DNA Polymerase (Takara Biomedical Technology Co., Ltd, Beijing, China) with specific primers (Table S1) and analyzed using Plant-Care (http://bioinformatics.psb.ugent.be/webtools/plantcare/html/) (accessed on 23 April 2020).

The dual-luciferase transient expression system was conducted according to the previous study [34]. To assess the transactivation activity of HuMYB1, full-length coding sequences of *HuMYB1* was inserted into the pGreenⅡ BD-62-SK as effectors. The reporter vector was modified from the pGreenII 0800-LUC vector [33]. Then, effectors of pGreenⅡ BD-62-SK-HuMYB1, pGreenⅡ BD-62-SK, pGreenⅡ BD-62-SK-VP16 (positive control) and reporter were separately transformed into *Agrobacterium tumefaciens* strain GV3101. The effector and reporter were infiltrated into *N. benthamiana* leaves with a mixture of 9:1, and LUC and REN activities were measured by dual-luciferase assay kit (Promega, Madison, WI, USA) after 72 h.

To assess the transcriptional effects of HuMYB1 on the promoters of *ADH1*, *CYP76AD1-1* and *DODA1*, these promoters were inserted into the pGreenII 0800-LUC vector as reporters, while *HuMYB1* were inserted into the pGreenII 62-SK vector as effectors. The reporter and effector were transformed into *Agrobacterium tumefaciens* strain GV3101 and then infiltrated into *N. benthamiana* leaves. LUC and REN activities were analyzed as above.

## 3. Results

### 3.1. Identification of R2R3-MYB Genes from H. undatus

The pitaya MYB encoding genes were identified by the alignment of the pitaya genome and MYB domain HMM profile (PF00249) using BLASTP software. A total of 231 candidate deduced amino acid sequences containing MYB or MYB-like repeats were obtained. The MYB domains were subsequently analyzed by Pfam and SMART. In total, 75 1R-MYB proteins, 105 R2R3-MYB proteins, four R1R2R3-MYB proteins and one 4R-MYB protein were obtained from the *H. undatus* genome (Table S2). Besides this, the theoretical pI and molecular weight ranged from 4.26 kDa (HuMYB137) to 10.96 kDa (HuMYB171) and 8.91 kDa (HuMYB143) to 213.32 kDa (HuMYB154), respectively (Table S2).

**Figure 1:**
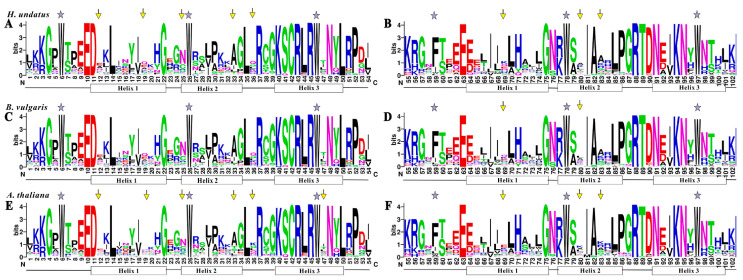
Comparison of DNA-binding domains of R2R3-MYB transcription factor in *H. undatus*, *B. vulgaris* and *A. thaliana*. Sequence logos of the R2 (**A**,**C**,**E**) and R3 (**B**,**D**,**F**) repeats are based on conserved alignments from *H. undatus* (**A**,**B**), *B. vulgaris* (**C**,**D**) and *A. thaliana* (**E**,**F**). The overall height of each stack indicates the conservation of the sequence at the position, whereas the height of letters within each stack represents the relative frequency of the corresponding amino acid. Highly conserved tryptophan (W) and phenylalanine (F) residues are indicated by asterisks. The positions with different patterns between *H. undatus*, *B. vulgaris* and *A. thaliana* are indicated by arrows.

To investigate the homologous domain sequence features, conservation and divergence of 105, 70 and 126 R2 and R3 repeats from *H. undatus* (betalain-producing plant), *B. vulgaris* (betalain-producing plant) and *A. thaliana* (anthocyanin-producing plant) were assessed using multiple alignment analyses (Figure 1). The results showed that the basic regions of pitaya, beet and Arabidopsis MYB domains (about 104 amino acid residues) were rarely deleted or inserted, as previously reported in the other plants [35,36], while they were widely divergent in the length and amino acid outside of the MYB domains. However, the length of Chinese cabbage and rice were larger than that of Arabidopsis based on the space between neighboring tryptophan (Trp, W) residues [37]. In pitaya, six highly conserved triplet W residues were located in R2 repeat (position 6, 26 and 46), R3 repeat (position 78 and 97) (Figure 1A,B) and the first Trp residue in R3 repeat (position 59) was replaced by a hydrophobic amino acid Phe (F). These conserved W (F) residues are indispensable in maintaining the helix-turn-helix structure of MYB domains [38]. Similar localization existed in both beet and Arabidopsis, suggesting the evolutionary conservation of MYBs among plants (Figure 1C,F). Besides this, the 3’ region of R2 domain in pitaya R2R3-MYBs contained a highly conserved LRPD motif. Compared to Arabidopsis, the positions at 12, 19, 25, 33 and 36 in R2 repeats and 69, 80 and 83 in R3 repeats showed difference in pitaya (Figure 1). Difference was detected between pitaya and beet in the position 69 and 80 in R3 repeats, indicating the divergence of the MYB domain between pitaya and beet was less than that of pitaya and Arabidopsis.

### 3.2. Genomic Location of HuMYBs

**Figure 2:**
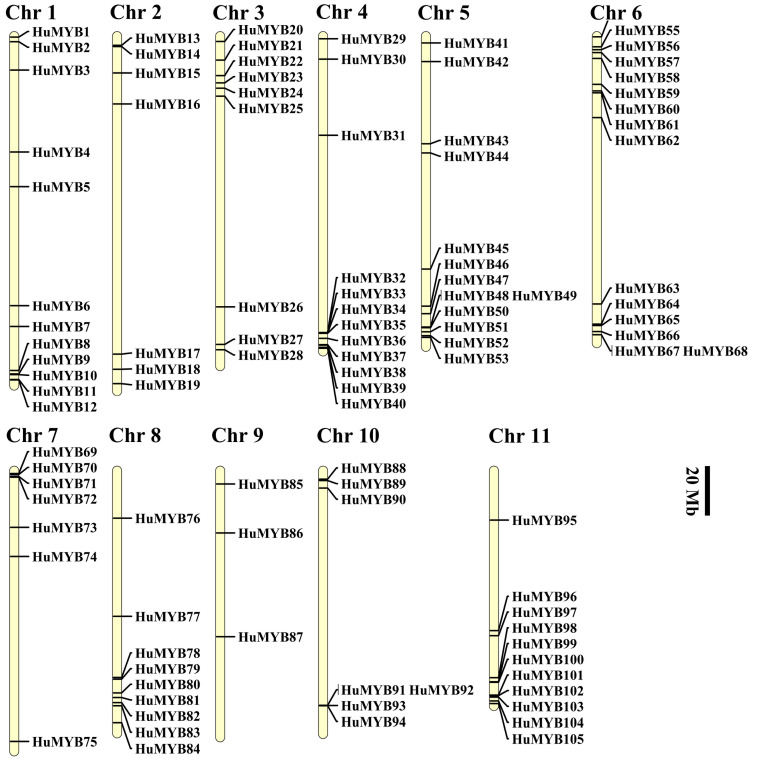
Distribution of R2R3-MYB members on the eleven chromosomes of pitaya. *R2R3-HuMYB* genes are shown on the right of each chromosome. Gene positions and the size of each chromosome can be estimated using the scale on the right of the figure, the scale indicates 20 megabases (Mb).

Genome chromosomal location analyses showed that all the 105 *R2R3-MYB* genes were distributed throughout all 11 chromosomes (Chrs) in the pitaya genome (Figure 2) and renamed as *HuMYB1* to *HuMYB105* according to their location in 11 Chrs. The largest number of *MYB* genes (fifteen) mapped on Chr 6, followed by thirteen on Chr 5. However, there were only three *HuMYBs* on Chr 9. In general, *MYB* genes are absent in the central sections of chromosomes including centromere and pericentromeric regions. Relatively high densities of *HuMYB* genes were observed at the end of Chr 3, 6 and 7 as well as the bottom of Chr 1, 4, 5, 6, 8 and 11 (Figure 2). The same uneven distributions in all chromosomes were also reported in the other plants, such as *A. comosus* [13], *Brassica rapa* [37] and *Eucalyptus grandis* [39].

### 3.3. Synteny Analyses of Pitaya R2R3-MYB Subfamily

**Figure 3:**
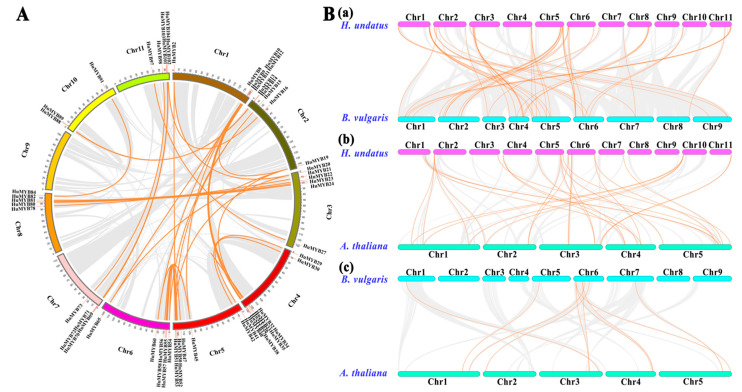
Synteny analyses of the pitaya, beet and Arabidopsis *R2R3-MYB* genes. (**A**) Schematic representation of interchromosomal relationships of the pitaya *R2R3-MYB* genes. (**B**) Gene duplication and synteny relationship of *R2R3-MYB* genes between pitaya and beet (**a**), pitaya and Arabidopsis (**b**), and beet and Arabidopsis (**c**). Gray lines in the background indicate the collinear blocks between genomes, while orange lines highlight the syntenic *MYB* gene pairs.

Gene duplication is recognized to occur throughout plant evolution and plays an important role in expanding the large gene families in plants [40]. The duplication patterns and synteny relationship of the *HuMYBs* were analyzed using BLASTP and MCScanX methods. In total, 40 segmental duplication events with 61 *HuMYBs* happened in the pitaya genome (Figure 3A; Table S3). *HuMYBs* were located within synteny blocks on almost all chromosomes except for Chr 9, indicating that *HuMYBs* underwent through expansion during genome evolution. If two or more *MYB* genes resided within 20 kb in the same chromosome, a tandem duplication event was defined [41]. In this study, three HuMYB tandem duplication pairs happened in Chr 5 (*HuMYB48* and *HuMYB49*), Chr 6 (*HuMYB67* and *HuMYB68*) and Chr 10 (*HuMYB93* and *HuMYB94*). These results suggested that large-scale segmental duplication events were the major contributors to the expansion of the *R2R3-MYB* gene family in *H*. *undatus*.

To further investigate the potential evolutionary mechanism of R2R3-MYB subfamily, we constructed the comparative syntenic maps of *H*. *undatus*, *B. vulgaris* and *A*. *thaliana* in pairs (Figure 3B). Finally, 46 collinear *R2R3-MYB* gene pairs between pitaya and beet (Figure 3B(a); Table S4-1), 28 between pitaya and Arabidopsis (Figure 3B(b); Table S4-2) and 10 between beet and Arabidopsis (Figure 3B(c); Table S4-3) were identified. The number of orthologous events of HuMYB-BvMYB was much more than that of HuMYB-AtMYB and BvMYB-AtMYB, indicating that the evolutionary distance between pitaya and beet was closer than that of pitaya and Arabidopsis. In addition, the area of all synteny blocks between pitaya and beet was far greater than that of pitaya and Arabidopsis. These results suggested that the evolutionary distance between pitaya and beet is closer than that of pitaya and Arabidopsis.

### 3.4. Phylogenetic, Gene Structure and Motif Composition Analyses of R2R3-MYBs

**Figure 4:**
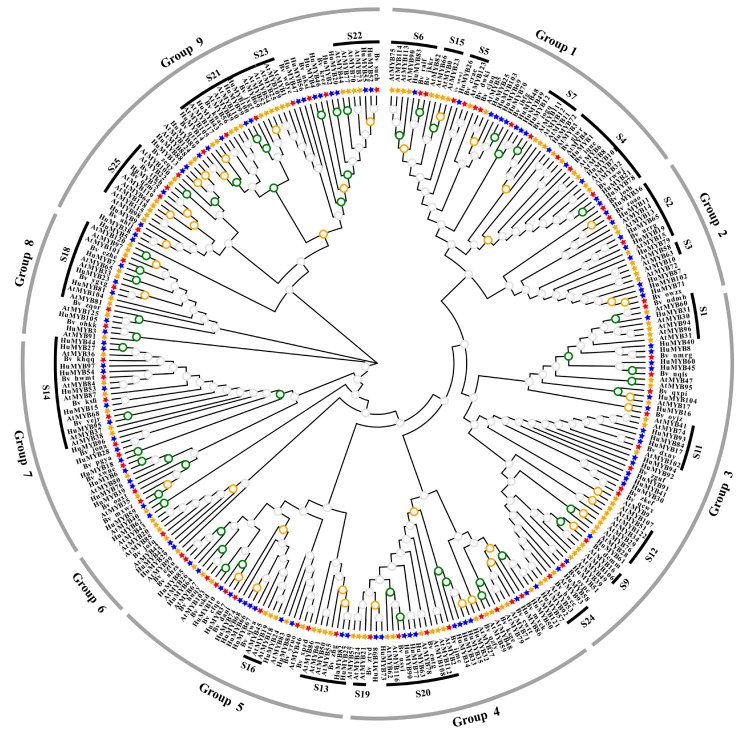
Phylogenetic Maximum likelihood (ML) tree (1000 bootstraps) with R2R3-MYB proteins from *H. undatus* (Hu), *B. vulgaris* (Bv) and *A. thaliana* (At) by MEGA7.0. Green circles indicate the bootstrap value range from 81 to 100 in the tree, orange is from 60 to 80, gray is from 0 to 59. Groups and Subgroups (according to the *A. thaliana*) are labeled with different alternating tones of gray and black background, respectively.

To explore the putative function of the 105 *HuMYBs*, functional groups were performed using *MYB* genes from *B. vulgaris* and *A. thaliana. A. thaliana* was chosen because *MYB* genes have been extensively studied in this model plant while *B. vulgaris* was a vital plant material for betalain studies. Most MYB proteins shared similar functions cluster in the same phylogenetic clades, suggesting that most closely-related MYBs could recognize similar target genes and possess redundant, overlapping and/or cooperative functions [42]. By the ML method, an evolutionary tree of 105 *HuMYBs*, 70 *BvMYBs* and 126 *AtMYBs* was constructed (Figure 4). In the classification of the *R2R3-MYB* genes, the subgroup (S) categories from *A. thaliana* were also labeled in the phylogenetic tree [7]. According to the phylogenetic tree topology, 105 HuMYB proteins were classified into nine groups (designated as Group 1 to Group 9). HuMYBs were distributed with AtMYBs and BvMYBs in most subgroups. However, species-specific AtMYBs were also detected in S10, S12 and S15 (clades without HuMYBs and BvMYBs).

*HpWRKY44* and *BvMYB1* are involved in betalain biosynthesis but they share high homology with anthocyanin regulated TFs including *AtWRKY44*, *AtMYB75*, *AtMYB90*, *AtMYB113* and *AtMYB114* [17,43]. Thus, there is probably a link of transcription regulation between betalains and anthocyanins. Ten R2R3-MYB genes in Group 1 attracted our attention due to their gathering in S4 to S7 from Arabidopsis and Bv_ihfg (Figure 4). Particularly, *HuMYB12* was clustered with *Bv_iogq* and *AtMYB111* in S7, which controlled flavonol biosynthesis [3,16]. *HuMYB83* was close to *Bv_ralf*, *Bv_jkkr* and *AtMYB90* in the S6 group, which is involved in betalain and anthocyanin biosynthesis [3,16,17]. *HuMYB26* was near *Bv_crae* and *AtMYB123* in S5, responsible for proanthocyanidins (PAs) biosynthesis in the seed coat of Arabidopsis [3]. In addition, five genes, i.e., *HuMYB1*, *HuMYB21*, *HuMYB72*, *HuMYB78* and *HuMYB101*, were gathered in S4 which encoded transcriptional repressors [3]. *HuMYB48* and *HuMYB49* were grouped with *Bv_ihfg* containing negative flavonoid regulator *FaMYB1* [16]. These results suggested that *HuMYB12*, *HuMYB26* and *HuMYB83* were candidate R2R3-MYB activators while *HuMYB1*, *HuMYB21*, *HuMYB48*, *HuMYB49*, *HuMYB72*, *HuMYB78* and *HuMYB101* were candidate transcriptional repressors.

**Figure 5:**
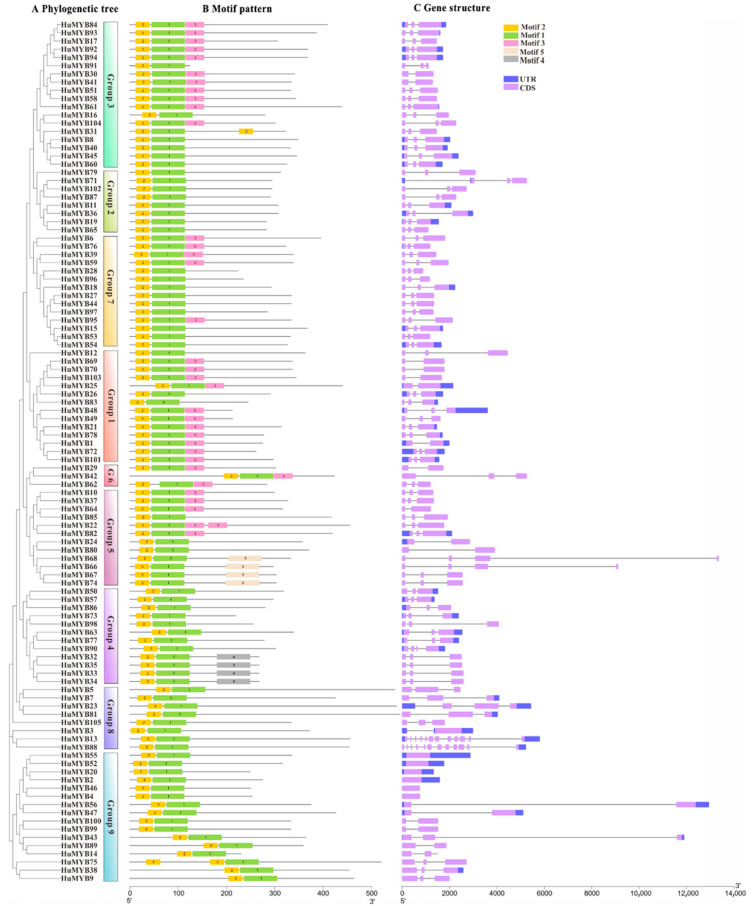
Phylogenetic relationship, gene structure and architecture of conserved protein motifs in *R2R3-MYB* genes from pitaya. (**A**) The neighbor-joining (NJ) tree on the left includes 105 R2R3-MYB proteins from pitaya with 1000 bootstrap. The MYB proteins were clustered into 9 groups. (**B**) Architecture of conserved protein motifs in 9 groups. Each motif is represented by a number on the colored box and their sequence were shown in Figure S1. (**C**) Exon–intron structures of R2R3-MYB proteins from pitaya. Exon(s) and intron(s) are represented by purple boxes and black lines, respectively. Untranslated region(s) are indicated by blue boxes.

Phylogenetic reconstruction of all 105 HuMYBs proteins were performed by the NJ method with 1000 bootstraps in MEGA7.0 (Figure 5A). The 105 HuMYBs were divided into nine groups, which was consistent with the result of the phylogenetic tree constructed by ML method in Figure 4. To understand the distribution of conserved motifs, the 105 R2R3-MYB protein sequences were analyzed by MEME software with five conserved motifs (named as motif 1 to 5) (Figure 5B and Figure S1). A total of 105 HuMYBs contained motifs 1, 2 and 3 which constituted R2 and R3 domains in the N-terminal, except for *HuMYB42* in Group 6 and *HuMYB9*, *HuMYB14*, *HuMYB38*, *HuMYB43*, *HuMYB75* and *HuMYB89* in Group 9, which were found in the central region. Besides this, motif 4 and motif 5 were respectively conserved in Group 4 and Group 5, which is consistent with the phylogeny analyses of 105 HuMYBs proteins.

The exon–intron structures of 105 R2R3-MYB coding sequences were analyzed (Figure 5C). Most R2R3-MYBs were clustered in the same group with the similar exon-intron structures, particularly with the same number of introns, such as Group 2 and Group 7. Except for six genes (*HuMYB2*, *HuMYB4*, *HuMYB20*, *HuMYB46*, *HuMYB52* and *HuMYB55*) in Group 9 without any intron, most of *HuMYBs* were disrupted by introns. In addition, more than 73% *HuMYBs* had three exons and two introns, which was also found in other plants [13,14].

### 3.5. Betalain Measurement and Expression Analyses of R2R3-MYBs during Pitaya Fruit Ripening

**Figure 6:**
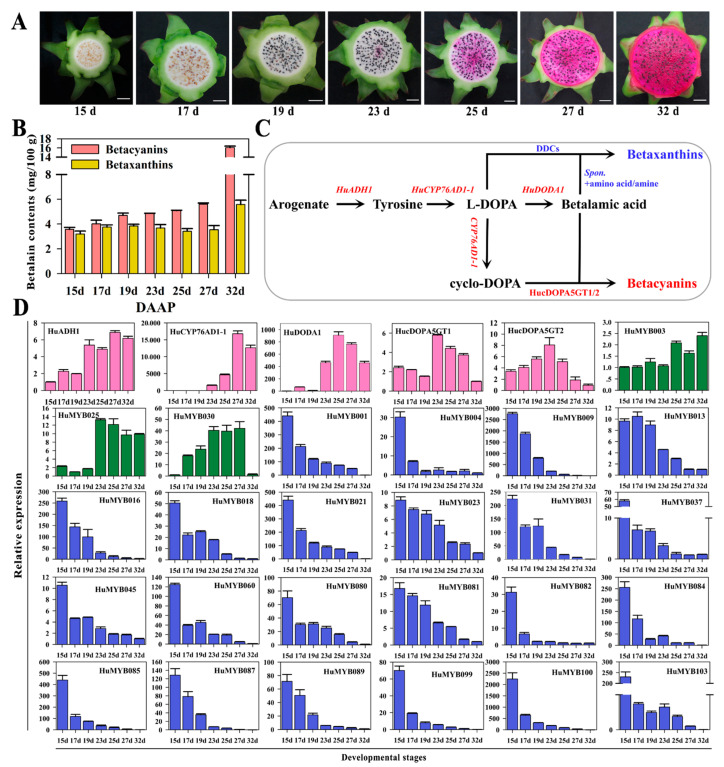
Expression analyses of *R2R3-MYB* genes in pulps during fruit ripening of ‘Guanhuahong’ pitaya. (**A**) Pulp coloration of fruit ripening of ‘Guanhuahong’ pitaya. Bars = 2 cm. (**B**) Contents of betacyanin and betaxanthin in pulps during fruit ripening of ‘Guanhuahong’ pitaya. DAF, day after flowering. (**C**) A brief pathway of betalain biosynthesis in pitaya. (**D**) Expression analyses of four structural genes of betalain biosynthesis pathway (labeled in pink), and candidate up-regulated (labeled in green) and down-regulated (labeled in blue) *R2R3-MYB* genes in pulps during fruit ripening of ‘Guanhuahong’ pitaya.

Betacyanin and betaxanthin contents were measured in seven ‘Guanhuahong’ pitaya fruit from 14th d to 32nd DAF (Figure 6A). The contents of betacyanins and betaxanthins in pulps increased throughout pitaya fruit ripening (Figure 6B). Betacyanin contents were increased gradually during fruit ripening and reached their maximum levels at the fully mature stage. *ADHα*, *CYP76AD1α*, *DODAα* and cDOPA5GT were essential structure genes involved in betalain biosynthesis (Figure 6C). Based on *H. undatus* genome data, homologous genes were obtained and renamed as *HuADH1* *(HU03G02979.1)*, *HuCYP76AD1-1* *(HU03G00480.1)*, *HuDODA1* *(HU03G01342.1)*, *cDOPA5GT1 (HU07G00239.1)* and *cDOPA5GT2 (HU03G00240.1)*. *HuADH1*, *HuCYP76AD1-1* and *HuDODA1* showed increasing trends and predominantly expressed in pulps of the later period (23rd to 32nd DAF) of ‘Guanhuahong’ pitaya (Figure 6D). *cDOPA5GT1* and *cDOPA5GT2* were predominantly expressed on the 23rd DAF when pulps started to accumulate betacyanins. The expression of the *HuADH1*, *HuCYP76AD1-1* and *HuDODA1* were closely related to betalain accumulation in pulps during fruit ripening of ‘Guanhuahong’ pitaya.

In order to analyze the expression levels of *R2R3-MYB* genes, qRT-PCR were performed to screen candidate *R2R3-MYB* genes related to betalain biosynthesis during fruit ripening of ‘Guanhuahong’ pitaya. As shown in Figure 6D, three *HuMYBs* (*HuMYB3*, *HuMYB25* and *HuMYB30*) showed upward trends while twenty-two *HuMYBs* (*HuMYB1*, *HuMYB4*, *HuMYB9*, *HuMYB13*, *HuMYB16*, *HuMYB18*, *HuMYB21*, *HuMYB23*, *HuMYB31*, *HuMYB37*, *HuMYB45*, *HuMYB60*, *HuMYB80*, *HuMYB81*, *HuMYB82*, *HuMYB84*, *HuMYB85*, *HuMYB87*, *HuMYB89*, *HuMYB99*, *HuMYB100* and *HuMYB103*) showed significant downward trends in pulps during fruit ripening of ‘Guanhuahong’ pitaya (Figure 6D). The qRT-PCR results were consistent with RNA-Seq data (PRJNA704510) that most *HuMYBs* were down-regulated in pulps during fruit ripening (17th, 23rd, 25th and 32nd) of ‘Guanhuahong’ pitaya (Figure S2). Additionally, fourteen genes were predominantly expressed on the 15th DAF when the seeds were white (Figure S3). Five genes were highly expressed on the 17th DAF when the seeds became red (Figure S3). Fourteen genes were predominantly expressed on the 19th DAF when seeds were black (Figure S3). Eleven genes were highly expressed on the 23rd DAF when the betacyanin started to accumulate in pulps (Figure S3). Twenty-two genes showed irregular trends during pitaya fruit ripening (Figure S4). These results suggested that many *HuMYBs* may function as repressors during pitaya fruit ripening.

### 3.6. Phylogenetic and Sequence Analyses of Pitaya 2R-MYB Repressors

**Figure 7:**
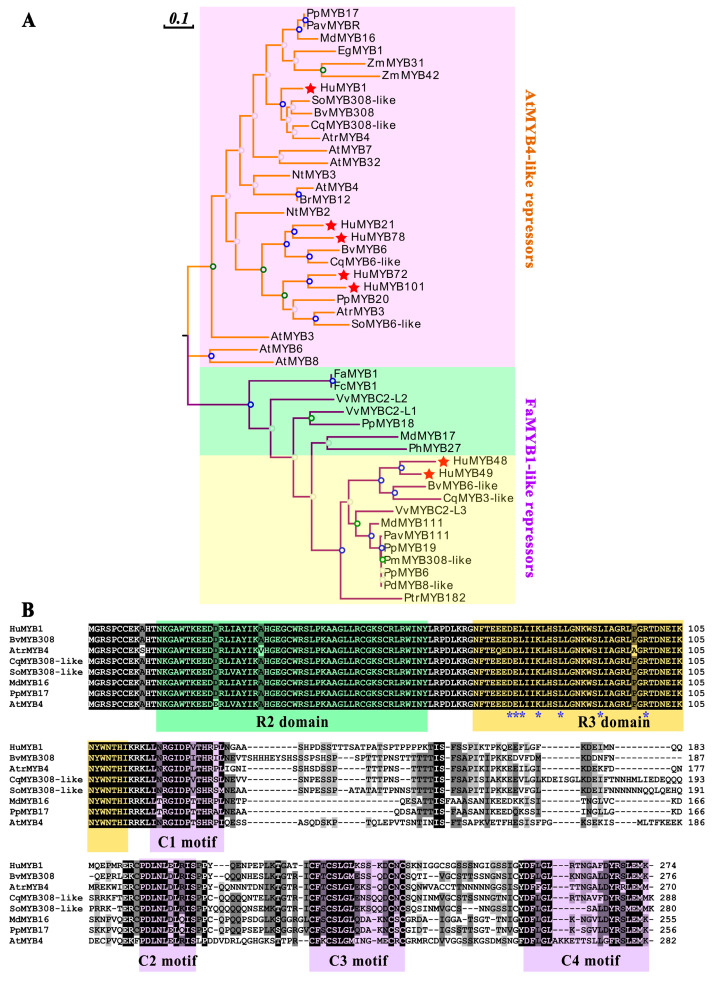
The sequence analyses of R2R3-MYB repressors. (**A**) The phylogenetic analyses of 2R-MYB repressor TFs based on the ML method with 1000 replications by MEGA7.0. Red background indicates the AtMYB4-like repressors while green and yellow background are anthocyanin and proanthocyanin repressors of FaMYB1-like repressors group, respectively. HuMYBs are labeled by the red star. (**B**) The alignment analyses of HuMYB1. R2 and R3 domains are labeled in green and orange shading, respectively. C1, C2, C3 and C4 motifs are labeled in purple shading. Blue asterisks indicate the motif ([D/E]Lx2[R/K]x3Lx6Lx3R) which can interact with a bHLH partner.

To further explore the R2R3-MYB repressors in pitaya, the phylogenetic relationship of seven HuMYBs, seventeen FaMYB1-like repressors and twenty-four AtMYB4-like repressors were constructed using the ML method with 1000 replications in MEGA7.0 (Figure 7A, Table S5). *HuMYB1* was first clustered with *AtrMYB4*, *SoMYB308-like*, *BvMYB308* and *CqMYB308-like* from betalain-producing plants, with *ZmMYB31* and *ZmMYB42*, which regulated phenylpropanoid genes in maize [44]. *HuMYB21* and *HuMYB78* were first grouped with *CqMYB6-like* and *BvMYB6*, and then with *NtMYB2* which repressed the regulation of anthocyanin biosynthesis [45]. *HuMYB72* and *HuMYB101* were clustered with *PpMYB20* which had repressive effects on the flavonoid pathway [46]. FaMYB1-like repressor group was divided into anthocyanin and proanthocyanin repressor subgroups [47]. *HuMYB48* and *HuMYB49* were grouped in proanthocyanin repressors. Both gathered first with *CqMYB3-like* and *BvMYB6-like* and then with *VvMYBC2-L3* which negatively regulated proanthocyanin biosynthesis [48]. These results suggested that seven *HuMYBs*, including five AtMYB4-like repressors (*HuMYB1*, *HuMYB21*, *HuMYB72*, *HuMYB78* and *HuMYB101*) and two FaMYB1-like repressors (*HuMYB48* and *HuMYB49*), were R2R3-MYB repressors.

Furthermore, the sequence alignment of the seven R2R3-MYB repressor proteins indicated that all of them had the conserved R2 and R3 domains in the N-terminal. Except for HuMYB21 and HuMYB78, the R3 domain of the other genes contained the bHLH binding motif ([D/E]Lx2[R/K]x3Lx6Lx3R) which was responsible for interaction with some bHLH TFs (Figure 7B, Figures S5 and S6). In addition to R2 and R3 domains, only HuMYB1 had four conserved motifs located in C-terminal in terms of C1 motif (KLIsrGIDPxT/SHRxI/L), C2 motif (pdLNLD/ELxiG/S or LxLxL), C3 motif (Cx1-2Cx7-12Cx2C) and C4 motif (FLGLx4-7V/LLD/GF/YR/Sx1LEMK) (Figure 7B). The C1 and C2 motifs were involved in bHLH interactions and considered as promoter repression domains [49,50]. AtMYB4-like repressors are characterized by C1 and C2 motifs in the C-terminal [51]. C4 motif was identified as negative regulation factor of floral volatile benzoid/phenylpropanoid compounds [52] and absent in FaMYB1-like repressors [53]. Besides this, in AtMYB4-like repressor group, HuMYB72, HuMYB78 and HuMYB101 only contained the C1 motif compared to HuMYB21 without conserved motif in C-terminal (Figure S5). In FaMYB1-like repressor group, HuMYB48 and HuMYB49 had the C1 and C2 motifs in their C-terminal (Figure S6). These results suggested that *HuMYB1*, *HuMYB21*, *HuMYB48*, *HuMYB49*, *HuMYB72*, *HuMYB78* and *HuMYB101* were R2R3-MYB TFs due to conserving in R2 and R3 domains in N-terminal. Five genes (*HuMYB1*, *HuMYB48*, *HuMYB49*, *HuMYB72* and *HuMYB101*) had a bHLH binding motif in R3 domain while six genes (*HuMYB1*, *HuMYB48*, *HuMYB49*, *HuMYB72*, *HuMYB78* and *HuMYB101*) had one to two conserved repression motifs in their C-terminal.

### 3.7. HuMYB1 Is a Nucleus-Localized Transcription Repressor

**Figure 8:**
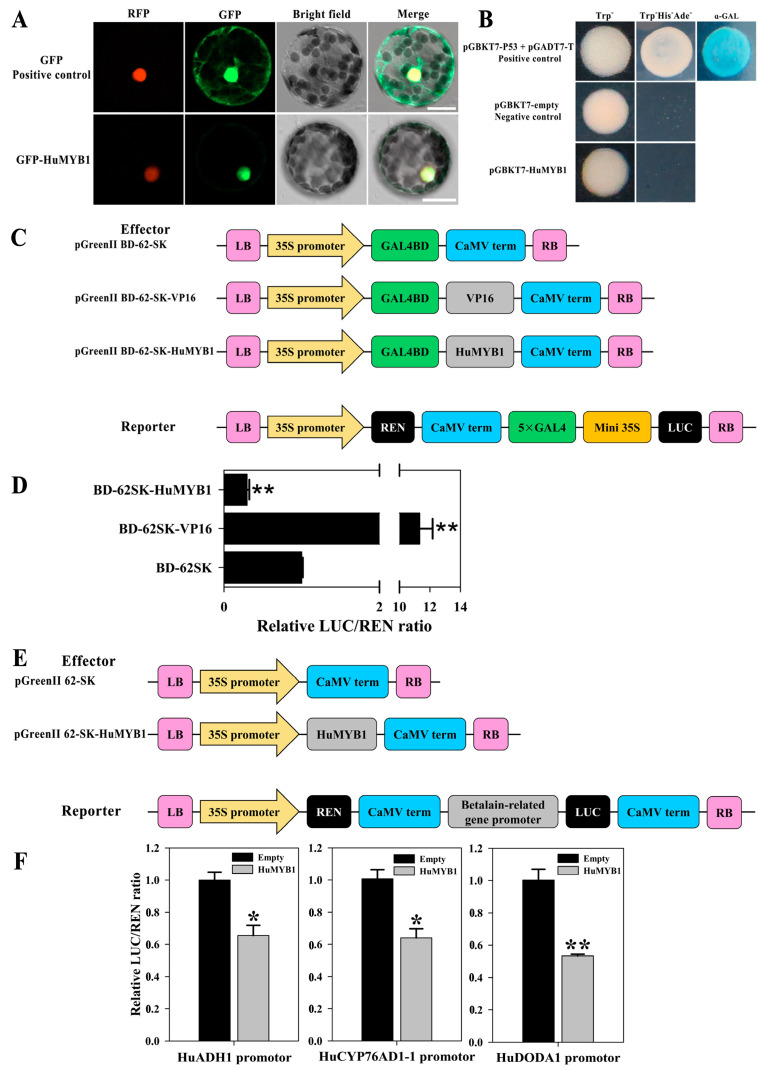
Subcellular localization and transcriptional activation analyses of *HuMYB1*. (**A**) Subcellular localization of *HuMYB1* in protoplasts of *Nicotiana benthamiana*. Bars = 20 μm. (**B**) Transcriptional activation of HuMYB1 in yeast cells. pGBKT7 and pGBKT7-53 + pGADT7-T were used as negative and positive control, respectively. (**C**) Diagrams of the reporter and effector vectors. (**D**) Transcriptional activation of *HuMYB1* in *N. benthamiana* leaves. The LUC/REN ratio of the empty BD-62SK vector was used as a calibrator (set as 1). BD-62SK-VP16 was used as a positive control. Asterisks represents highly significant differences at *p* value < 0.01 using two-tailed *t*-test, compared to BD-62SK. (**E**) Diagrams of the reporter and effector vectors. (**F**) *HuMYB1* inhibited the transcription of betalain-related genes including *HuADH1*, *HuCYP76AD1-1* and *HuDODA1* by dual-luciferase transient expression assay in *N*. *benthamiana* leaves. The ratio of LUC/REN of the empty vector (62SK) plus promoter was used as a calibrator (set as 1). Asterisks represents highly significant differences at *p* value < 0.05 (one asterisks) and *p* value < 0.01 (two asterisks) using two-tailed *t*-test, compared to 62SK.

*HuMYB1* probably plays an essential role in suppressing betalain biosynthesis in pitaya according to the above evolutionary, expression and sequence alignment analyses. To investigate the subcellular localizations of *HuMYB1*, the full-length coding sequence of *HuMYB1* was fused with the *GFP* to construct 35S-HuMYB1-GFP vectors which were transiently transformed into *N. benthamiana* leaves. The nuclear signals were captured in protoplasts after overexpressing *HuMYB1-GFP*, whereas *GFP* alone showed cytoplasmatic and nuclear signals (Figure 8A).

Full-length coding regions of *HuMYB1* were fused with the GAL4BD in the pGBKT7 vector to study the transcriptional activation abilities of HuMYB1 in yeast cells. As shown in Figure 8B, the transformed yeast cells of positive control (pGBKT753 + pGADT7-T) grew well in SD/-Trp-His-Ade and showed α-Gal activity, while yeast cells containing pGBKT7 (negative control) or pGBKT7-HuMYB1 did not, suggesting that HuMYB1 has no transactivation activities in yeast cells and probably functions as a transcriptional repressor in gene regulation.

The transcriptional repressor activities of HuMYB1 were confirmed in *N*. *benthamiana* leaves using the dual-luciferase reporter system (Figure 8C). Compared to the expression of negative control (BD-62SK), the expression of the positive control (BD-62SK-VP16) resulted in a higher value of LUC/REN ratio while the BD-62SK-HuMYB1 showed a significantly lower value (Figure 8D). It is consistent with the result from yeast cells. These results demonstrated that HuMYB1 was a nucleus-localized transcriptional repressor.

### 3.8. HuMYB1 Inhibited the Transcription of Three Betalain Biosynthesis-Related Genes

*HuADH1*, *HuCYP76AD1-1* and *HuDODA1* promoters were cloned from ‘Guanhuahong’ pitaya and the conserved *cis*-element motifs were predicted in Plant-CARE database. MYB binding sites were present in the promoters of *HuADH1*, *HuCYP76AD1-1* and *HuDODA1* (Table S6). Transient dual-luciferase assays in *N*. *benthamiana* leaves were performed to study whether HuMYB1 acted as the transcriptional repressor of *HuADH1*, *HuCYP76AD1-1* and *HuDODA1* (Figure 8E). As shown in Figure 8F, compared to the empty control (pGreenII 62-SK), LUC/REN ratio was significantly reduced when HuMYB1 was co-transformed with HuADH1 or HuCYP76AD1-1 or HuDODA1 pro-LUC reporter. These results indicated that HuMYB1 participated in betalain biosynthesis by repressing the transcription of betalain biosynthesis-related *HuADH1*, *HuCYP76AD1-1* and *HuDODA1* genes (Figure 8F).

## 4. Discussion

Although the genome-wide of MYB superfamily has been extensively studied in various plants, the identification and characterization of R2R3-MYB TFs based on the whole genome sequence of *H*. *undatus* were not reported yet. In our study, a total of 185 *MYB* genes (75 *1R-MYB*, 105 *R2R3-MYB*, four *R1R2R3-MYB* and one *4R-MYB*) were identified by genome-wide search and distributed in all *H. undatus* Chrs (Figure 2). The R2R3-MYB proteins are the largest subfamily which is consistent with the findings in Arabidopsis [3], pineapple [13] and *Salvia miltiorrhiza* [36]. Compared with beet, the number of pitaya R2R3-MYB members (105) is fewer than Arabidopsis (126) [3] but more than beet (70) [16], indicating R2R3-MYB subfamilies in pitaya and Arabidopsis were expanded. Low tandem and high segmental duplications resulted in the expansion of the *R2R3-MYB* gene family in *H*. *undatus*, which also occurred in cotton [54] and pineapple [13]. Based on the comparison of DNA-binding domains (Figure 1), synteny (Figure 3) and phylogenetic relationship (Figure 4) between pitaya, beet and Arabidopsis, closer genetic distance was detected between betalain-producing plants of pitaya and beet compared to pitaya and the anthocyanin-producing plant Arabidopsis [27].

**Figure 9:**
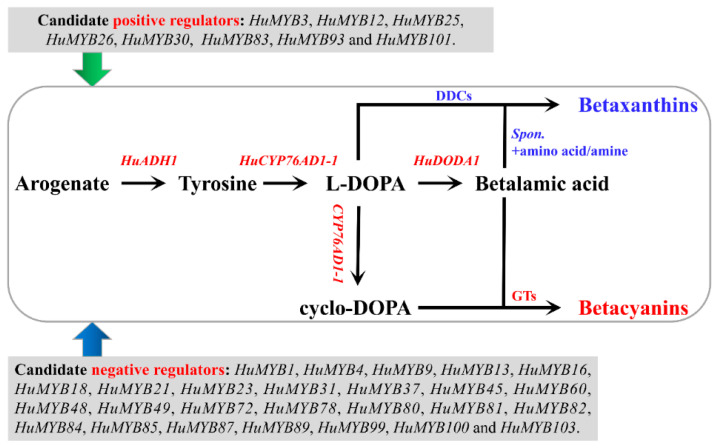
A hypothetical model of candidate *R2R3-MYB* genes involved in betalain biosynthesis of pitaya. *Spon.* indicates spontaneous.

Among 105 *R2R3-MYB* genes, expression patterns of 91 *HuMYBs* were analyzed in pulps of the seven fruit ripening stages of ‘Guanhuahong’ pitaya. Some genes showed preferential expressions on the 15th, 17th, 19th or 23rd DAF, suggesting that they play multiple regulatory roles during pitaya fruit ripening (Figure S3). *HuMYB3*, *HuMYB25* and *HuMYB30* shared a similar expression pattern with betalain biosynthesis-related structure genes of *HuADH1*, *HuCYP76AD1-1* and *HuDODA1*. However, 20.9% *R2R3-MYB* genes kept downward trends and were contrary to the expressions of *HuADH1*, *HuCYP76AD1-1* and *HuDODA1* during pitaya fruit ripening (Figure 6). Additionally, *HuMYB93* and *HuMYB101* shared similar expression patterns with *HucDOPA5GT2* involved in the formation of betanin (Figure S3). ADH and ADT (arogenate dehydratase) can catalyze arogenate to form tyrosine and phenylalanine, respectively, and then form betalains and anthocyanins [55]. In the present study, *HuMYB83* was clustered with *BvMYB1* and *AtMYB90*. *BvMYB1* is responsible for betalain biosynthesis [17], while *AtMYB90* is involved in anthocyanin biosynthesis [3]. The transcription regulation of betalains and anthocyanins may exist a connection in their evolutionary relationship. Thus, *HuMYB12*, *HuMYB26* and *HuMYB83* may be involved in betalain biosynthesis since they were clustered with Arabidopsis subgroups 7, 5 and 6 involved in the regulation of flavonoid biosynthesis. Besides this, five AtMYB4-like repressors (*HuMYB1*, *HuMYB21*, *HuMYB72*, *HuMYB78* and *HuMYB101*) and two FaMYB1-like repressors (*HuMYB48* and *HuMYB49*) were identified according to the phylogenetic tree. In total, eight positive regulators and twenty-seven negative regulators were obtained by expression profiles and phylogenetic analyses (Figure 9).

According to expression and sequence analyses, *HuMYB1* showed a downward trend during fruit ripening (Figure 7) and was closer to *AtMYB4*, which negatively regulated phenylpropanoid pathway [3]. Besides this, *HuMYB1* acted as a nucleus-localized transcriptional repressor involved in betalain biosynthesis pathway by repressing the transcription of *HuADH1*, *HuCYP76AD1-1* and *HuDODA1* (Figure 8)*. BvMYB1* is involved in betalain biosynthesis by targeting the *CYP76AD1* and *DODA* promoter while *HpWRKY44* has a functional role in the regulation of *CYP76AD1* [17,43]. Thus, HuMYB TFs are probably involved in betalain biosynthesis by binding to the *HuADH1*, *HuCYP76AD1-1* and *HuDODA1* promoters in pitaya. Therefore, a hypothetical model of betalain biosynthesis in pitaya was proposed according to these candidate *HuMYBs* possibly involved in betacyanin biosynthesis by regulating the expression levels of *HuADH1*, *HuCYP76AD1-1*, *HuDODA1* and/or GTs (Figure 9). Besides this, ABRE, E-box, G-box and W-box *cis*-elements were detected in the sequences of *HuADH1*, *HuCYP76AD1-1* and *HuDODA1* promoters, which were the binding sites of TFs such as AREB/ABF, bZIP, bHLH and WRKY [43,56,57,58]. Therefore, these HuMYB TFs are possibly involved in betalain biosynthesis in pitaya. Moreover, the transcription activity of MYB proteins was influenced by the interaction with other TFs, such as bZIP, bHLH, NAC and WRKY TFs [59,60,61,62]. The activity of MYBs and bHLHs appears to be regulated by cytosolic WD40 repeat proteins by forming highly dynamic MBW complexes. WRKY can activate the transcription of anthocyanin-related genes by engaging with the MBW complex to form MBWW complex. Thus, further studies need to be carried out to determine whether the other TFs such as AREB/ABF, bZIP, bHLH and WRKY are involved in betalain biosynthesis, or HuMYB1 can coordinate with the other TFs to regulate betalain biosynthesis.

## 5. Conclusions

In conclusion, this study is the first report on identification and characterization of *R2R3-MYB* gene family based on the genome of *H. undatus*. A total of 105 *R2R3-MYB* genes were obtained from the *H. undatus* genome. These R2R3-MYB genes were distributed in all 11 chromosomes with conserved R2 and R3 repeats and expanded its subfamily members through segmental duplication with a closer distance of beet than Arabidopsis. They were functionally classified into nine groups, consisting of conserved motif and exon–intron clustering. Phylogeny, sequence alignment and expression patterns of seven candidate R2R3-MYB repressors were further analyzed. *HuMYB1* was grouped in the AtMYB1-like repressor group with bHLH binding motif and C1-C4 motifs. Besides this, *HuMYB1* showed a downward expression trend opposite to betalain accumulation in pulps during fruit ripening of ‘Guanhuahong’ pitaya. HuMYB1 was a nucleus protein and could reduce the transcription activity of *HuADH1*, *HuCYP76AD1-1* and *HuDODA1* with transcription repression activities. The present study provides valuable information for a better understanding of MYB TFs involved in fruit ripening and betalain biosynthesis in pitaya.

## Data Availability

The data presented in this study are openly available in the NCBI PRJNA704510.

## Supplementary Materials

The following are available online at https://www.mdpi.com/article/10.3390/cells10081949/s1, Figure S1: Consensus sequences of the group specific motifs. Figure S2: The heatmap of *R2R3-MYB* genes in pulps of ‘Guanhuahong’ pitaya. Figure S3: The expression analyses of *R2R3-MYB* genes in pulps during fruit ripening of ‘Guanhuahong’ pitaya. Figure S4: The expression analyses of *R2R3-MYB* genes in pulps during fruit ripening of ‘Guanhuahong’ pitaya. Figure S5: The sequence analyses of HuMYB21, HuMYB72, HuMYB78 and HuMYB101. Figure S6: The sequence analyses of HuMYB48 and HuMYB49. Table S1: Summary of primers used in this study. Table S2: Summary of MYB members information in pitaya genome. Table S3: Segmentally and tandemly duplicated *HuMYB* gene pairs. Table S4: One-to-one orthologous relationships between pitaya, beet, and Arabidopsis. Table S5: Proteins used in Figure 7. Table S6: The regulatory motifs found within the *HuADH1*, *HuCYP76AD1-1*, and *HuDODA1* promoter.
