# Supplementary material for: Genome-Wide Characterization of R2R3-MYB Transcription Factors in Pitaya Reveals a R2R3-MYB Repressor HuMYB1 Involved in Fruit Ripening through Regulation of Betalain Biosynthesis by Repressing Betalain Biosynthesis-Related Genes
Source: Cells. 2021 Jul 31;10(8):1949. doi: 10.3390/cells10081949 (PMC8391165; doi:10.3390/cells10081949)
Supplement: Supplementary file 1 [file cells-10-01949-s001.zip › cells-1279019 supplementary proofreading back/Supplementary Files/Supplement figures - Copy.pdf]

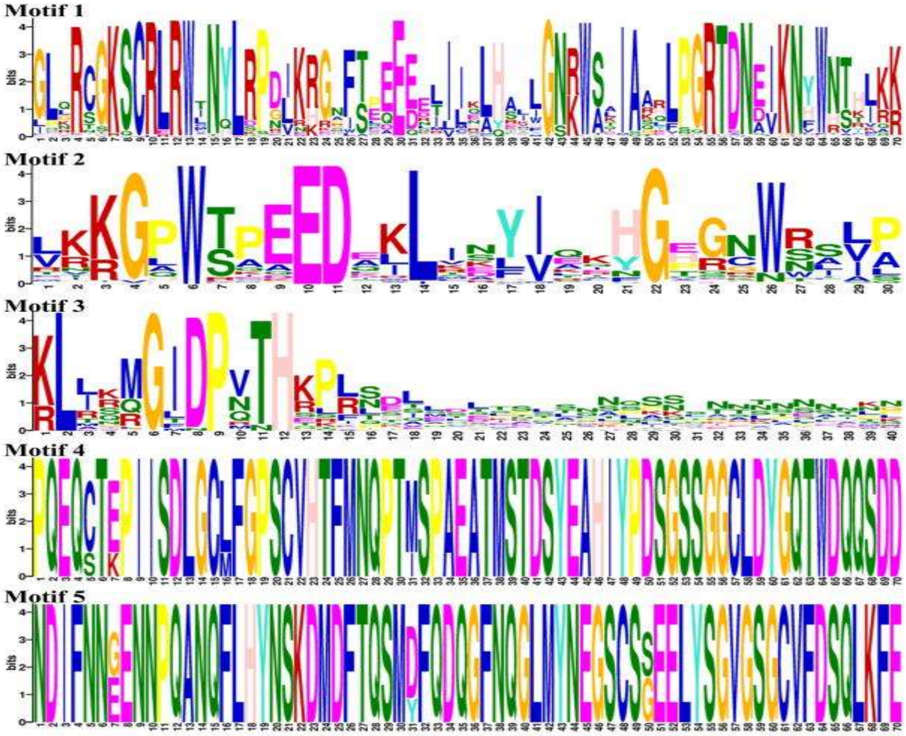

Figure S1. Consensus sequences of the group specific motifs.

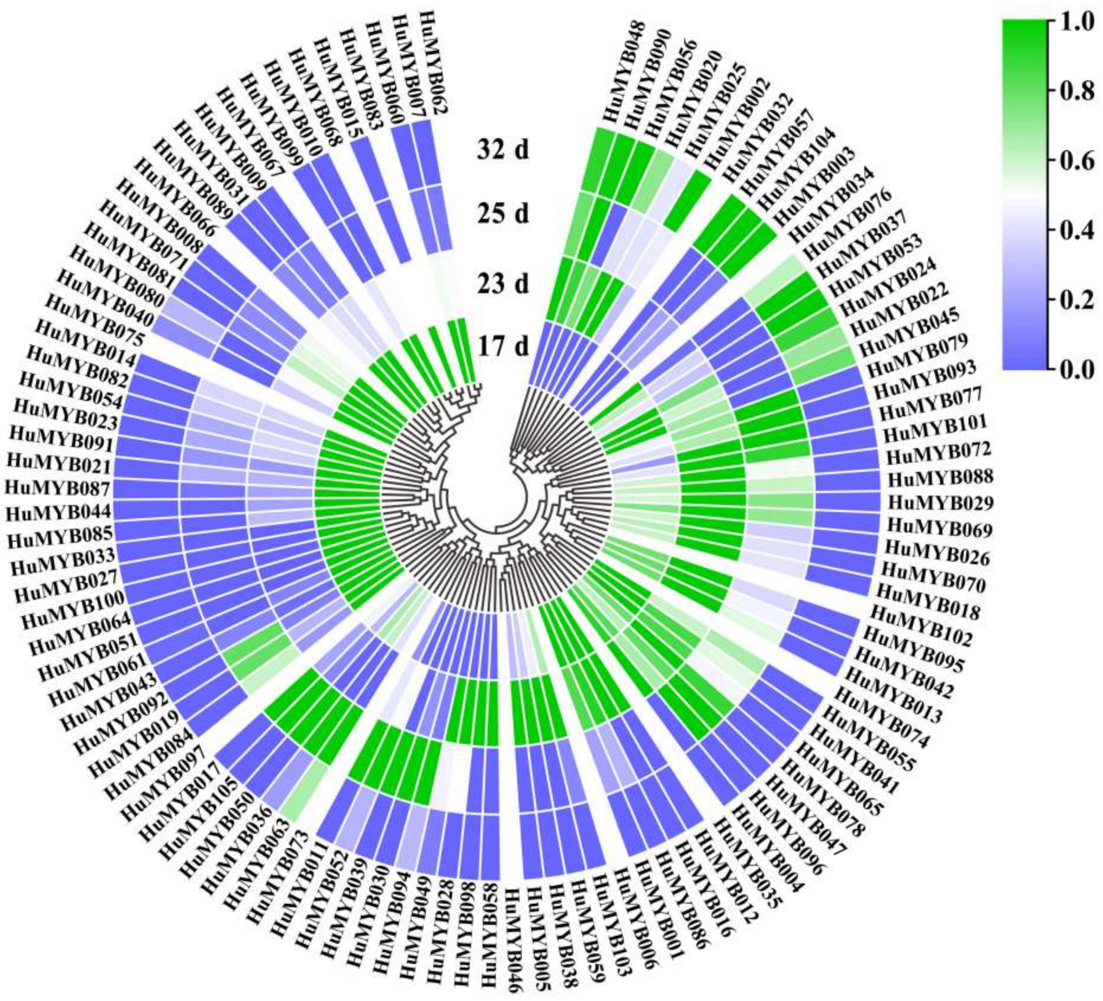

Figure S2. The heatmap of *R2R3-MYB* genes in pulps of 'Guanhuahong' pitaya.

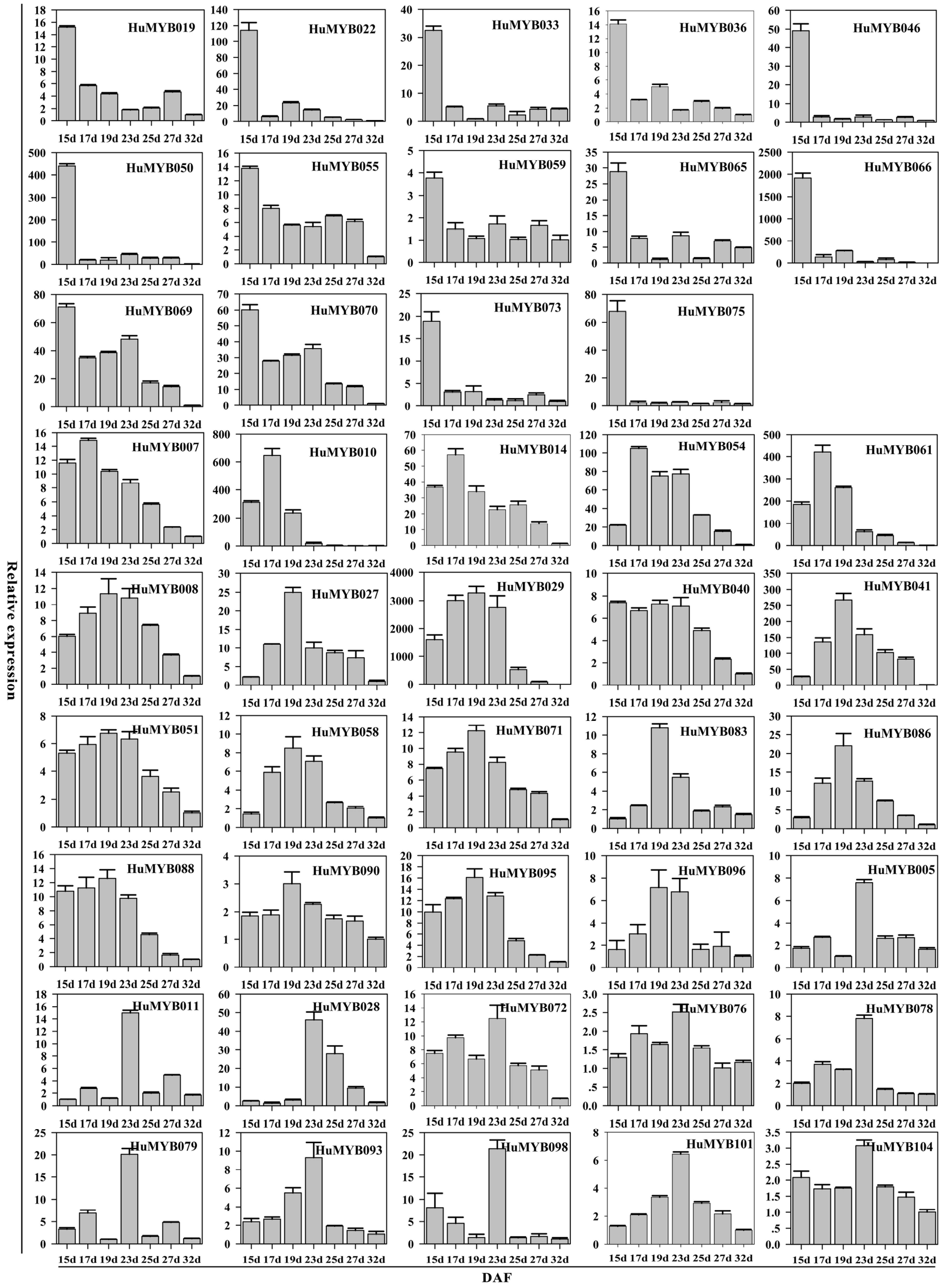

Figure S3. The expression analyses of *R2R3-MYB* genes in pulps during fruit ripening of 'Guanhuahong' pitaya.

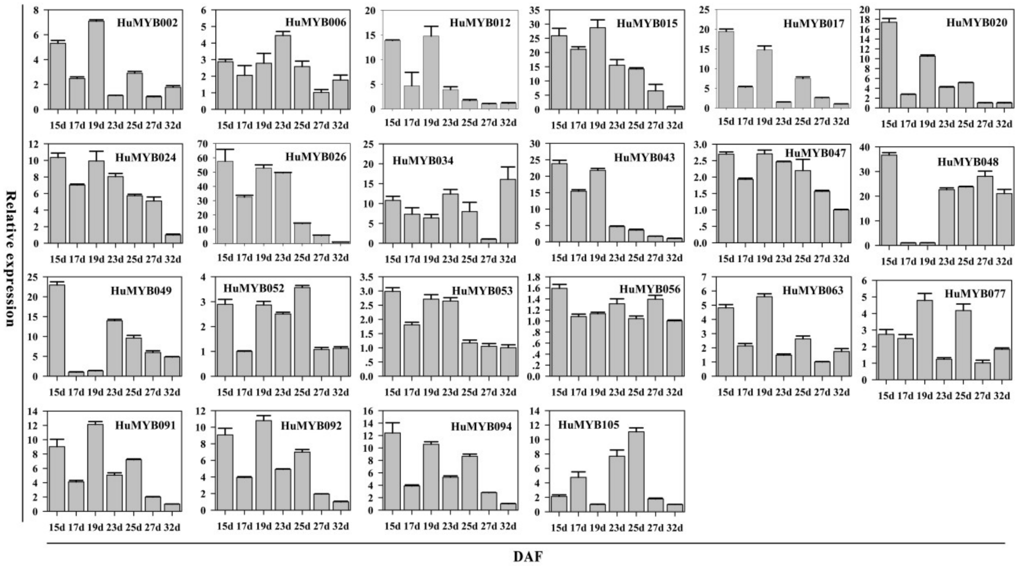

Figure S4. The expression analyses of *R2R3-MYB* genes in pulps during fruit ripening of 'Guanhuahong' pitaya.

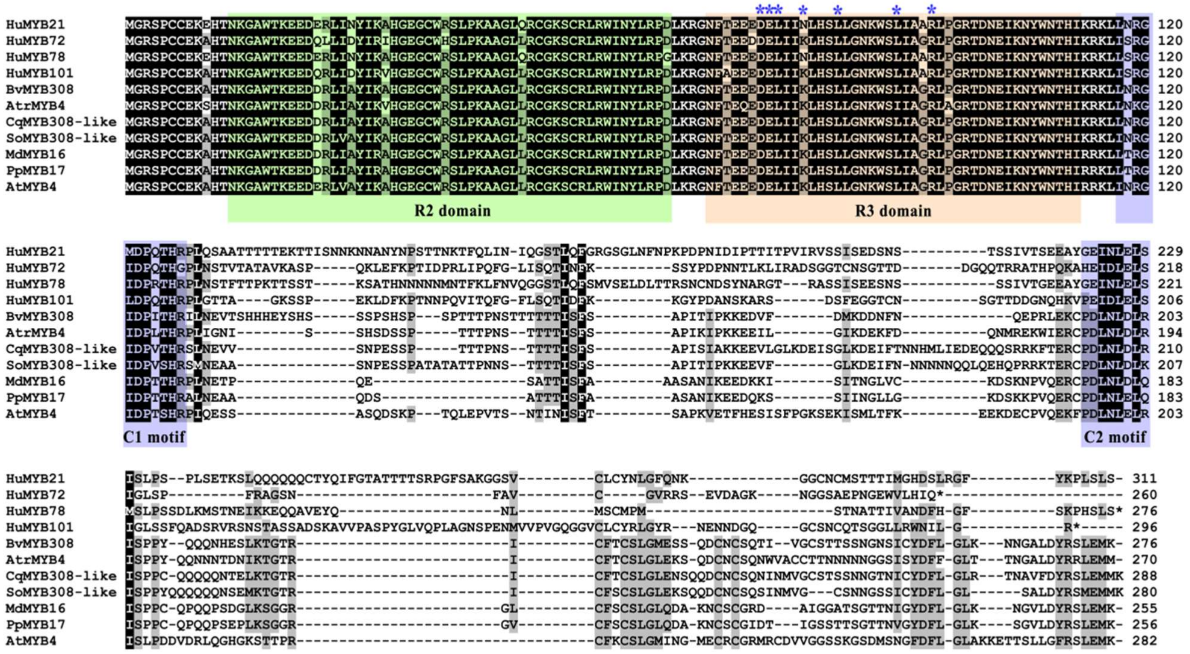

Figure S5. The sequence analyses of HuMYB21, HuMYB72, HuMYB78 and HuMYB101. R2 and R3 domain were labeled in green and orange shading, respectively. C1 and C2 motif were labeled in purple shading. Blue asterisks indicated the motif ([D/E]Lx2[R/K]x3Lx6Lx3R) which allows to interact with a bHLH partner.

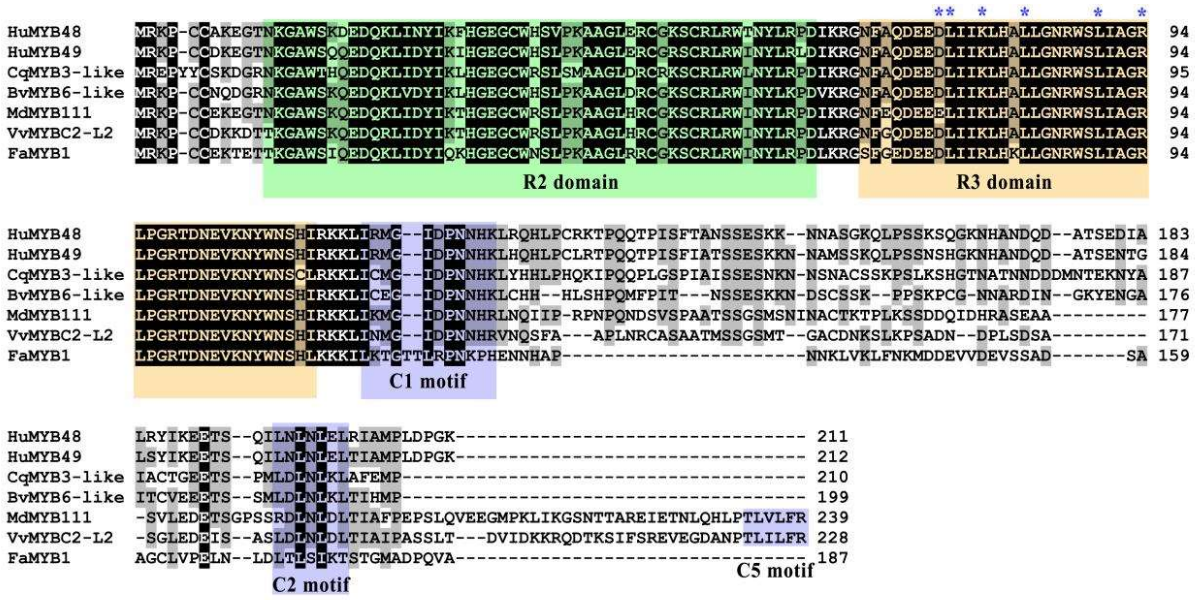

Figure S6. The sequence analyses of HuMYB48 and HuMYB49. R2 and R3 domain were labeled in green and orange shading, respectively. C1, C2 and C5 motif were labeled in purple shading. Blue asterisks indicated the motif ([D/E]Lx2[R/K]x3Lx6Lx3R) which allows to interact with a bHLH partner.
