# Supplementary material for: Genome-Wide Characterization of R2R3-MYB Transcription Factors in Pitaya Reveals a R2R3-MYB Repressor HuMYB1 Involved in Fruit Ripening through Regulation of Betalain Biosynthesis by Repressing Betalain Biosynthesis-Related Genes
Source: Cells. 2021 Jul 31;10(8):1949. doi: 10.3390/cells10081949 (PMC8391165; doi:10.3390/cells10081949)
Supplement: Supplementary file 1 [file cells-10-01949-s001.zip › cells-1279019 supplementary proofreading back/Supplementary Files/Table S1.pdf]

Table S1 Summary of primers used in this study

Assay	Primer names	Sequences (5'-3')
RT-qPCR	HuADH1-F	TGATGATGGTGAGAAGACGA
	HuADH1-R	GTAGTCGGAGCGAGAGTAGG
	HuCYP76AD1-1-F	CAAAGTGGTTCTTGGGAAAC
	HuCYP76AD1-1-R	GGTCTCAGCCTTACGAGGT
	HuDODA1-F	TGTGCCTTCTGTATCTGCCG
	HuDODA1-R	TGTCTTGAACCCTCCTGCTA
	HucDOPA5GT1-F	GAGAGTGGTGGAGTTGGTGT
	HucDOPA5GT1-R	CTACGGAAAATAACCGCATC
	HucDOPA5GT2-F	CGGCGTATTTGTCACTGT
	HucDOPA5GT2-R	CGACCCATCAGCGTATCTTA
	HuMYB1-F	GCCCTCCCTATCAAGAAAAT
	HuMYB1-R	AATGCTGCTTCCAATACCAT
	HuMYB2-F	GCCAGAAAAGAGAATGGAGTTC
	HuMYB2-R	GGCAAGGACAGAGTCAAAGC
	HuMYB3-F	GACAAACCTCTAACCCAGACC
	HuMYB3-R	GGTGCCCTTCCTCTAACTCT
	HuMYB4-F	AGCGACAATGCTGACTTTGA
	HuMYB4-R	CTCCTCTTCTCTGCCCACAC
	HuMYB5-F	ACTGGTCGGTGTTGATTGTG
	HuMYB5-R	GCCATCTCTCTGCTCTCGTC

HuMYB6-F	ATTGACAGGGATGGGAATCG
HuMYB6-R	TTCTTGGTGAGGAGGTGGAG
HuMYB7-F	CCACATCCACAAACACCTCA
HuMYB7-R	AACAGTGCGACCCAGAAAAG
HuMYB8-F	CGTGCCGTTCCCACTAATAC
HuMYB8-R	TGCCCATCTGTTGCCTAAA
HuMYB9-F	GGACCCCTTTGATAACTCCA
HuMYB9-R	GAATGCCCTAAAAACCCACA
HuMYB10-F	TCACTATGGGGTTGGCTGTT
HuMYB10-R	TGGTGGAGATGAATGATGAGA
HuMYB11-F	GACGCACCGACAATGAAATC
HuMYB11-R	CCTCCAGAACATCACCCTTT
HuMYB12-F	GCAAGATGAAGGGATTTCTC
HuMYB12-R	TGATGATGAAGCAGTTGAGC
HuMYB13-F	AAGGTTGCTGCTTTGATGC
HuMYB13-R	GTTCCTCGCTGTCTTTGCTC
HuMYB14-F	GGTTTCCCTTTCTCATCATCC
HuMYB14-R	CCCCACATTCTTGTTTTTGC
HuMYB15-F	TCGGTCTCTTCGCTACCTATG
HuMYB15-R	CCTTCACCATCCAAAATGCT
HuMYB16-F	CGATTCAAAGGCAATGTCAA
HuMYB16-R	CCATAGGCGAAGGAAGTGAT

HuMYB17-F	TCCCAACTTCCCACTTCAAC
HuMYB17-R	AAATCCTGTTTGCCCCAAG
HuMYB18-F	GAGGAGGTGGTGAAGGAATA
HuMYB18-R	AGGGTGAAGGTGTAGAGGTG
HuMYB19-F	GTCGTCCTCGTCCCAAGAT
HuMYB19-R	CACATCCAACCAAAAATCCA
HuMYB20-F	ACGCTAAGTTCGGCAACAAG
HuMYB20-R	TAGACAATCTCCCGCAATCG
HuMYB21-F	GCCTCCTTGGTAATAAATGG
HuMYB21-R	TGGTGGTTTTCTCGGTAGTT
HuMYB22-F	AAGGAGGCATAGACCCCAAT
HuMYB22-R	GACGGTTTGTCGGAGTTTTT
HuMYB23-F	GCCTCCTTGGTAATAAATGG
HuMYB23-R	TGGTGGTTTTCTCGGTAGTT
HuMYB24-F	TGAGACGGTTGTTGAGGTTG
HuMYB24-R	CAAAAGGAAAGGCGACATCT
HuMYB25-F	AACCAGCCACTTCAACAACT
HuMYB25-R	TCATCCCACTGACCTCCTAC
HuMYB26-F	GCTTTTAGGCAACAGATGGT
HuMYB26-R	TTAGTGGTGGAGGAACTGGT
HuMYB27-F	CCATTTAGCAATCACCACCA
HuMYB27-R	TACCCTTCTGGCTTCACCAT

HuMYB28-F	ACATCAATCTCCCCAGCATT
HuMYB28-R	ATCCAAAACCCATCTTCGTG
HuMYB29-F	GCGAGGACTTCTTACCAATGA
HuMYB29-R	TTTTTGATGTGGGTGTTCCA
HuMYB30-F	TCTTCTTCACCCGAGGCTAA
HuMYB30-R	GCCACCATTTCTGTCATCAAG
HuMYB31-F	GCCACCATCATTCTGCTAT
HuMYB31-R	TTCTCGCTGTCATTGTTGTT
HuMYB33-F	ATTCAGGGAGGCTTTGTGG
HuMYB33-R	CTCGGTGGACTGCTCTTGA
HuMYB34-F	TGGAGAATGAGTGGGGAAAA
HuMYB34-R	GGCAAGACGAGCAATGAAGT
HuMYB36-F	AAGGTGTCTGGAAAGGGTGA
HuMYB36-R	GTGGGGAAACTGGCTCAAT
HuMYB37-F	TTGGAGAGGGAAAACTGTGG
HuMYB37-R	AGGGAAACCCAGCGATAGTG
HuMYB40-F	AACTCATCGTCGTCAACATCC
HuMYB40-R	CGCAGGAGGCGTAAGTATTC
HuMYB41-F	CCAGGAAGGACAGACAATGAA
HuMYB41-R	TTTGGTGGGGTGGAAAGTAG
HuMYB43-F	TTTGGACCCCTTCTCCTTCT
HuMYB43-R	CACAGTTTCGTAGCCCCATT

HuMYB45-F	GAACTGGCTCAAACGGAAGA
HuMYB45-R	CACAAAATGGCTTGCTCTCA
HuMYB46-F	TAGCGACAATGCTGACTTTGA
HuMYB46-R	TCTCCTCTTCTCTGCCCACA
HuMYB47-F	AGCCTCCCACACAGAGAATA
HuMYB47-R	CCATCAGTTTAGTCCGTCCT
HuMYB48-F	CACCGATTTCATTCACTGC
HuMYB48-R	TGGGATGTTTCTTCCTTGAT
HuMYB49-F	GGAAGGACAGACAACGAAGT
HuMYB49-R	CAGATGAAGTCGCAATGAAT
HuMYB50-F	CATCAGAGCGACGAAAATCC
HuMYB50-R	TGGTTGGTTGGGAAACTTGT
HuMYB51-F	GGAAGAGCAGGCAGTGAGAC
HuMYB51-R	GAATGATGAGGGGAGGGAGT
HuMYB52-F	GTGTCTGTCTCTGCCTGGAT
HuMYB52-R	CTCATTCCTCACCATCTCCT
HuMYB53-F	CCACCCACAGAATACGATGTT
HuMYB53-R	GTGCTGCTTTTGCCTATGTC
HuMYB54-F	GGTCTATCATCGCCACACAA
HuMYB54-R	AAACTGCTGCCTTCCCTTTT
HuMYB55-F	ACAACGCCATCAAGAACCAC
HuMYB55-R	GCTCAAACCCGAGACATTC

HuMYB56-F	GCCTCCTTCCTTTTCCAATC
HuMYB56-R	ATCTCCGTCCTTCTGGTTGA
HuMYB58-F	ACCCTTGAGACCCCAGATTT
HuMYB58-R	GGTGGAGATGGAGAGGAAGA
HuMYB59-F	TGACTCCTCTGCTGTTGCTG
HuMYB59-R	CCATAACTTGGGCTCCTGAA
HuMYB60-F	CCAGAGAGGACAGACAACGA
HuMYB60-R	GAGATAATGGGGACGACGAA
HuMYB61-F	ATAAGTCCTCGTTGGGGTCA
HuMYB61-R	TTCCTCGTCACCGTCTTGAT
HuMYB63-F	ATGCCAAGCAACTCAAATGT
HuMYB63-R	GTGGTGGAGGTTCCGATAGA
HuMYB65-F	GGAAACCATCATTCACCTTCA
HuMYB65-R	CCATTTTGGACTTTGGCTCT
HuMYB66-F	GCAAAGGAATGGAAAGAGTTGT
HuMYB66-R	TGCCTAAAAAGCGATGAAGG
HuMYB69-F	CAAAGGGAAGGCACGAAAG
HuMYB69-R	AGGTGGGATGATGGAGGTAA
HuMYB70-F	CAAAGGGAAGGCACGAAAG
HuMYB70-R	AGGTGGGATGATGGAGGTAA
HuMYB71-F	CAAGGCAGGAGTGAAGAAGG
HuMYB71-R	AACCCATCGTAAGCGACAAC

HuMYB72-F	TTGGCACTCTCTCCCTAAAG
HuMYB72-R	AATCTCCCAGCAATCAAGG
HuMYB73-F	CATCTGAAGCACAGCCAAAG
HuMYB73-R	GGAAGGGAAATAAGGGTGGT
HuMYB75-F	CCATTTCGGACACCAATACC
HuMYB75-R	ATGTTGAGAAGAGGGGACGA
HuMYB76-F	ACCTAATGGCGGAGATTGC
HuMYB76-R	AAGAAGTGCTGGGTTGTTGG
HuMYB77-F	TGGACGGTAGAGGAAGACCT
HuMYB77-R	CGAACATCAGGACGCAAATA
HuMYB78-F	GTGAAGGTTGTTGGAGGTCT
HuMYB78-R	TGTGGAGGTTGATGATGAGT
HuMYB79-F	AAGAACAAGAAAGGGCGAAA
HuMYB79-R	GAGGGTTGAGGGGATGATG
HuMYB80-F	TGTTGATGGGTTGGTCTCTG
HuMYB80-R	TTGTTGCTGTTAGTGCTTCG
HuMYB81-F	CCATAGTCGTGTTGGAAGCA
HuMYB81-R	TGAGGAGGGTTGGTTGATGT
HuMYB82-F	CATCCATCACCCGTTTCAT
HuMYB82-R	AGAGCCGTTGTTGCTTTCAT
HuMYB83-F	GGTGCTATTCAACCCTATGC
HuMYB83-R	TCCTCCAACTCAAAGTCCAT

HuMYB84-F	AGAGGGTTTGGTGAATGTCG
HuMYB84-R	GCTTTGATGTTGTCCACTCG
HuMYB85-F	CGTCCCTAAACTTGCTGGTC
HuMYB85-R	GAGCCCATCTGTTGCCTAAA
HuMYB86-F	ACCTCAAGCAAGGCAAGAGA
HuMYB86-R	TCTGTATTCCACAAGCCATCC
HuMYB87-F	TGTTACGATGCGGGAAAAGT
HuMYB87-R	CTGGCAAATGAGAAGCAATCT
HuMYB88-F	CGAGGACATTGGAGTAGCAG
HuMYB88-R	CGGGATTGTAGATGCCAGAG
HuMYB89-F	GCACGGCTAATCATCTCCAT
HuMYB89-R	TGAAAGAACCCAGGAAAAGC
HuMYB90-F	GCCTCATCTCCTTCTTCACC
HuMYB90-R	TTTACAGTTTTGCCCCTTGC
HuMYB91-F	GGACATCAAGAGGGGAAGATT
HuMYB91-R	TCAATACCCATTCGGAGGAG
HuMYB92-F	GCCAGGAAGGACTGATAACG
HuMYB92-R	TGGGAGTTCAAGAGGGAGGT
HuMYB93-F	GCCACTTCACTCCTCTGCTC
HuMYB93-R	GTTTGTATCGGGGCTTGGAT
HuMYB94-F	TGGCAACCTCAACTTTCTCC
HuMYB94-R	CAATGACCGACGCACAAG

	HuMYB95-F	TTGGAAGCAGGTGGTCAATA
	HuMYB95-R	TTGGTGGTTTTGGTGTTGG
	HuMYB96-F	GAAGATACTGGGAGGCAACG
	HuMYB96-R	TGAGGTGGGCATTGTTCAT
	HuMYB98-F	CCAAACACCTCCCAAGACAA
	HuMYB98-R	GAGTTCCAAACACCTTCACCA
	HuMYB99-F	CTCCATCTTGTGGGTCGTCT
	HuMYB99-R	TCATTGTCGTCGTCTTCTGG
	HuMYB100-F	CTCCATCTTGTGGGTCGTCT
	HuMYB100-R	TCATTGTCGTCGTCTTCTGG
	HuMYB101-F	CACACCAACAAAGGAGCAT
	HuMYB101-R	AATCCAACGAAGCCTACAAC
	HuMYB103-F	ACTGGAACACCCACCTCAAC
	HuMYB103-R	TTGTGCTTTGCTTCTCTTGGT
	HuMYB104-F	GGAAGATTGCCTTGCTGTGA
	HuMYB104-R	CCTGCTTGCTTAGGGAGAGA
	HuMYB105-F	CATTCCCAACCTCCAAACAG
	HuMYB105-R	TCCCAAACAGGACAGTAGCC
Full-length cloning	HuMYB1-F	ATGGGGAGGTCCCCTTGTTGTG
	HuMYB1-R	TCATTTCATTTCTAAGCTTCTGTAATC
	HuMYB23-F	ATGGGAAGATCACCATGTTGT
	HuMYB23-R	TCATGATAAACTAAGAGGTTTGTAAAACC

	HuMYB48-F	ATGAGGAAGCCATGCTGT
	HuMYB48-R	TTATTTCCCAGGATCCAAAGGC
	HuMYB49-F	ATGAGGAAGCCATGCTGTGAT
	HuMYB49-R	TTATTTCCCAGGATCCAAAGGCAT
	HuMYB72-F	ATGGGACGCTCCCCTTG
	HuMYB72-R	CTATTGTATATGAAGAACCCATTCGC
	HuMYB78-F	ATGGGCAGATCACCGTGC
	HuMYB78-R	TCATGATAAACTATGAGGTTTGG
	HuMYB101-F	ATGGGACGTTCACCTTGC
	HuMYB101-R	TCATCGGCCTAATATATTCCACCT
Promoter isolation	HuADH-F	ATTTTCGCCCCTTTTTTTGGATTGATAAG
	HuADH-F	TGGTTGGGTGGAGGTTGGTGGAAATG
	HuCYP76AD1-F	AATTAGTGCAGAGGTATAATACTTGCATG
	HuCYP76AD1-F	GTTGGGCTATCCATGCTTTTGGGAAG
	HuDODA-F	ACGGCGGCGGCGATGACGATAACAACAAG
	HuDODA-R	TCTTTGCTTCTGGTGCTGCTGGTGCAAT
Subcellular localization	HuMYB1-GFP-F	GTCGACGGTATCGATAAGCTTATGGGGAGGTCCCCTTGTTGTG
	HuMYB1-GFP-R	TTTACTCATACTAGTGGATCCTTTCATTTCTAAGCTTCTGTAATCA
Y2H assay	HuMYB1-BD-F	CATGGAGGCCGAATTCATGCATGGGGAGGTCCCCTTGTTGTG
	HuMYB1-BD-R	GCCGCTGCAGGTCGACGTCATTTCATTTCTAAGCTTCTGTAATCA
Dual-luciferase	HuMYB1-BD -62SK-F	CGCCGTCTAGAACTAGTGGATCCATGGGGAGGTCCCCTTGTTGTG
	HuMYB1-BD -62SK-R	TCGATAAGCTTGATATCGAATTCTCATTTCATTTCTAAGCTTCTGTAATCA

expression assay	HuMYB1-62SK-F	GGCCGCTCTAGAACTAGTGGATCCATGGGGAGGTCCCCTTGTTGTG
	HuMYB1-62SK-R	ATCGATAAGCTTGATATCGAATTCTTTCATTTCTAAGCTTCTGTAATCA
	HuADH-pro-0800-F	TATAGGGCGAATTGGGTACCATTTTCGCCCCTTTTTTTGGATTGATAAG
	HuADH-pro-0800-R	TTGGCGTCTTCCATGGTGGTTGGGTGGAGGTTGGTGGAAATG
	HuCYP76AD1-pro-0800-F	TATAGGGCGAATTGGGTACCAATTAGTGCAGAGGTATAATACTTGCATG
	HuCYP76AD1-pro-0800-R	TTGGCGTCTTCCATGGGTTGGGCTATCCATGCTTTTGGGAAG
	HuDODA-pro-0800-F	TATAGGGCGAATTGGGTACCACGGCGGCGGCGATGACGATAACAACAAG
	HuDODA-pro-0800-R	TTGGCGTCTTCCATGGTCTTTGCTTCTGGTGCTGCTGGTGCAAT
