# Supplementary material for: Genome-Wide Characterization of R2R3-MYB Transcription Factors in Pitaya Reveals a R2R3-MYB Repressor HuMYB1 Involved in Fruit Ripening through Regulation of Betalain Biosynthesis by Repressing Betalain Biosynthesis-Related Genes
Source: Cells. 2021 Jul 31;10(8):1949. doi: 10.3390/cells10081949 (PMC8391165; doi:10.3390/cells10081949)
Supplement: Supplementary file 1 [file cells-10-01949-s001.zip › cells-1279019 supplementary proofreading back/Supplementary Files/Table S5.pdf]

Table S5 Proteins used in Figure 7

Gene	Species	Family	GenBank ID
AtMYB3	*Arabidopsis thaliana*	Brassicaceae	BAA21618.1
AtMYB4	*Arabidopsis thaliana*	Brassicaceae	BAA21619.1
AtMYB6	*Arabidopsis thaliana*	Brassicaceae	EFH48703.1
AtMYB7	*Arabidopsis thaliana*	Brassicaceae	AEC06531.1
AtMYB8	*Arabidopsis thaliana*	Brassicaceae	Q9SDS8.1
AtMYB32	*Amaranthus tricolor*	Amaranthaceae	AEE86444.1
AtrMYB3	*Amaranthus tricolor*	Amaranthaceae	AVI04856.1
AtrMYB4	*Amaranthus tricolor*	Amaranthaceae	AVI04857.1
BvMYB6	*Beta vulgaris*	Amaranthaceae	XP_010675151.1
BvMYB6-like	*Beta vulgaris*	Amaranthaceae	XP_010693797.1
BvMYB308	*Beta vulgaris*	Amaranthaceae	XP_010690379.1
BrMYB12	*Brassica rapa*	Brassicaceae	ADZ98868.1
CqMYB3-like	*Chenopodium quinoa*	Amaranthaceae	XP_021754972.1
CqMYB6-like	*Chenopodium quinoa*	Amaranthaceae	XP_021742109.1
CqMYB308-like	*Chenopodium quinoa*	Amaranthaceae	XP_021775509.1
EgMYB1	*Eucalyptus gunnii*	Myrtaceae	CAE09058.1
FaMYB1	*Fragaria × ananassa*	Rosaceae	AAK84064.1
FcMYB1	*Fragaria chiloensis*	Rosaceae	ADK56163.1
MdMYB16	*Malus domestica*	Rosaceae	ADL36756.1
MdMYB17	*Malus domestica*	Rosaceae	ADL36757.1

MdMYB111	*Malus domestica*	Rosaceae	ADL36754.1
NtMYB2	*Narcissus tazetta*	Amaryllidaceae	ATO58377.1
NtMYB3	*Narcissus tazetta*	Amaryllidaceae	AGO33166.1
PavMYB11	*Prunus avium*	Rosaceae	ALH21142.1
PavMYB111	*Prunus avium*	Rosaceae	ALH21138.1
PavMYBR	*Prunus avium*	Rosaceae	ADY15315.1
PhMYB27	*Petunia hybrida*	Solanaceae	AHX24372.1
PdMYB8-like	*Prunus dulcis*	Rosaceae	XP_034208432.1
PpMYB6	*Prunus persica*	Rosaceae	XP_007215900.1
PpMYB17	*Prunus persica*	Rosaceae	ALO81020.1
PpMYB18	*Prunus persica*	Rosaceae	ALO81021.1
PpMYB19	*Prunus persica*	Rosaceae	ALO81022.1
PpMYB20	*Prunus persica*	Rosaceae	ALO81023.1
PtrMYB182	*Populus tremula*	Salicaceae	AJI76863.1
SoMYB6-like	*Spinacia oleracea*	Amaranthaceae	XP_021847942.1
SoMYB308-like	*Spinacia oleracea*	Amaranthaceae	XP_021838682.1
VvMYBC2-L1	*Vitis vinifera*	Vitaceae	NP_001268133.1
VvMYBC2-L2	*Vitis vinifera*	Vitaceae	NP_001268180.2
VvMYBC2-L3	*Vitis vinifera*	Vitaceae	AIP98385.1
ZmMYB31	*Zea mays*	Panicoideae	CAJ42202.1
ZmMYB42	*Zea mays*	Panicoideae	CAJ42204.1
